# Mercy Hospital

**Published:** 1875-12

**Authors:** Roswell Park

**Affiliations:** A. M., Interne.


### Hospitals.

### MERCY HOSPITAL.

### SERVICE OF PROF. HOLLISTER.

(Reported by ROSWELL PARK, A.M., Interne.)

*Melanotic Cancer on the Arm—Metastasis to the Liver—Death—Autopsy.*

W. H. H., male, æt. 40, admitted Oct. 9th, 1875. Patient stated that a year or so ago he first noticed a little "black wart" on his right arm, at the insertion of the deltoid. Last March this, having increased in size, was examined by a physician of this city, and pronounced a melanotic cancer. The medical gentleman advised its removal by caustic paste, and, with consent of patient, proceeded to so remove it, employing the cautery during several successive visits. About four weeks ago he noticed that his urine was becoming highly colored; at the same time he began to feel a soreness or lameness in the back, and fever set in, with evening exacerbations.

On admission: the patient of an anxious cast of countenance, well nourished, pulse 100, skin warm and moist; the seat of the cancer, with the cicatrix consequent upon the operation, considerably pigmented, and the whole showing how and where the conical arrows had been introduced for its removal. In the right axilla are two enlarged glands, one as large as a hen's egg. Percussion gives a dull note over the greater part of the right side of the abdomen, the region of dullness extending from three inches above the crest of the ilium to a line two inches above the normal limit of the liver. Palpation reveals a præternatural hardness over the same region. The urine is normal in quantity, but high colored; and the application of proper tests shows the presence of biliary coloring matter. The tongue is coated—white in the middle—brown toward the edges. The stomach somewhat irritable; bowels pretty regular; appetite poor. Patient has some cough and complains of pain, especially at night with the fever, and of insomnia.

He was put upon the use of a mixture containing ammon. mur. and syr. glycyrrhizæ, with quiniæ sulph., gr. vij. twice a day, and tr. opii camph. in drachm doses, *pro re nata*, and with a view of stimulating the secretions he was ordered the pil. cath. comp. as many as required.

Besides, morphia had to be given at night to procure sleep. The bowels were rather costive until Nov. 2nd, when an enema produced two bloody evacuations; and since then, the bowels were loose and the stools contained more or less blood. The œdema of the lower extremities, slight on admission of the patient, gradually increased and extended to the genitals. In the beginning of November he several times vomited some stercoraceous matter, lost his appetite entirely, became occasionally delirious, and failed rapidly; on Nov. 9th, he sank into a stupor and died. An hour before his death an extensive extravasation of blood took place beneath the ocular conjunctiva.

*Autopsy—six hours after death.* External appearances: marked effusion of blood under the conjunctivæ; evidences of capillary stasis everywhere, especially lower part of right leg. Body much emaciated.

Lungs and heart normal in appearance. Liver enormously enlarged, weighing nearly fifteen pounds, and extending up to the right nipple, displacing the right lung and the mediastinum. It presented a nodular appearance, and, upon section, showed masses of melanotic deposit of all sizes, from that of a pin head to that of a walnut. Gall bladder normal. Kidneys and spleen slightly enlarged and congested, but without deposit; nor could any deposit be found in the alimentary tract. In the right iliac vein a thrombus was found—extending under Poupart's ligament, and occluding its lumen. The tissues of the abdomen and lower extremity were densely infiltrated with serum. The pigment of the enlarged axillary glands was noticeable through the skin. The brain was not examined.

